# Editorial: Eastern Philosophies and Psychology: Towards Psychology of Self-Cultivation

**DOI:** 10.3389/fpsyg.2017.01083

**Published:** 2017-06-29

**Authors:** Kwang-Kuo Hwang, Yung-Jong Shiah, Kin-Tung Yit

**Affiliations:** ^1^Psychology Department, Kaohsiung Medical UniversityKaohsiung, Taiwan; ^2^Counseling Psychology and Rehabilitation Counseling, National Kaohsiung Normal UniversityKaohsiung, Taiwan; ^3^Institute of Philosophy, Center for General Education, National Sun Yat-sen UniversityKaohsiung, Taiwan

**Keywords:** Eastern Philosophies, Buddhism, Confucianism, Taoism, self-cultivation, culture-inclusive theiries, multiple-philosophical paradigms

Based on rich experience of developing Chinese indigenous psychology in Taiwan for more than 30 years, Hwang proposed a unique epistemological strategy for constructing culture-inclusive theories of psychology which consists of two steps: First, he constructed a *Mandala model* of self (Hwang, 2011, 2015a) and a *Face and Favor* model of social interaction (Hwang, 1987, 2012). Because these two models are supported to be universal, the second step of his strategy is using them as frameworks to analyze any cultural system in opposition to the pan-cultural dimensional approach of reductionism prevalent in mainstream psychology (Hwang, 2015b).

In his book *Self-exertion and Conscience: Getting out of Weberian maze* (Hwang, 2015c), he criticized the fallacy of Eurocentrism and the fallacy of conflation committed in Max Weber's (1864–1920) famous works *Religious in China: Confucianism and Taoism* (Weber, 1951), and he used this strategy to analyze pre-Qin Classics of Confucian for studying its morphostasis. He then traced the morphogenesis of Confucianism during its major progress in the history of China and Japan respectively (Hwang, 2015c).

Because Confucian ethics and morality are supposed to be the transcendental formal structure for Chinese people's life world, once it has been clearly identified, the formal structure can be utilized to construct a series of culture-inclusive theories with a careful consideration of its manifestations in varies contexts of social interaction (Hwang, 2016). In order to demonstrate the usefulness of this approach, Hwang (2012) constructed a series of theoretical models on social exchange, moral judgments, face dynamism, achievement motivation, and conflict resolution in his book *Foundations of Chinese Psychology*.

For the sake of establishing an autonomous academic tradition of social science in Confucian culture, we have organized a research team to promote this movement in Taiwan. Hwang encourages his followers to utilize this approach to construct their own theoretical models for conducting empirical research in Chinese society.

Highly impressed by their remarkable performances, we decided to increase the visibility of our research team by publishing their works in an international journal of high reputation, and *Frontiers in Psychology* became our first choice. We called for paper on *Philosophical and Theoretical Psychology* from international academic community and obtained total submission of 22 abstracts. Eventually 11 articles had been accepted for publication after a strict procedure of review in accordance with standards of FIP.

Following Hwang's (2015a,b) strategy, Wu conceptualized the process of cultivating the ideal self in Confucian education on the basis of her cultural-semantic analysis of the “*Lessons for Learning*” (Xue-Ji) in the Classic of Rites (Liji). Fwu et al. studied the mediating role of self-exertion on the effects of effort on learning virtues and emotional distress after academic failure; Chen et al. examined high-school teachers' beliefs about effort and their attitudes toward struggling and smart students. Findings of both empirical researches can be used to illuminate Wu's theoretical analyses on the importance of education in Confucian society.

Huang proposed a dynamic model interpersonal harmony and conflict from a yin-yang perspective. Han studied the feeling of having or losing face in maintaining one's psychosocial equilibrium. Chen and Hwang extended the idea of personal face to national face in Confucian society. Hsu and Hwang proposed a tentative theory on the cognitive process of *yuanfen* to illustrate the idea of serendipity in relationship. All those empirical researches and theoretical analysis indicated the potentiality for the future development of Confucian theory of self-cultivation.

Chien's article reflected his own change from the trait approach of personality to the construction of culture-inclusive theory and showed the productivity of the later for future research on Chinese authoritarian orientation. Liu et al. tried to develop a virtue existential model of career development by integrating Western career theories of modernism and post modernism with the eastern wisdom of *I-Ching* (Book of Changes). Shiah made the first attempt to propose the Non-self Theory based on Buddhist teachings, as well as three ways for execution of the self-cultivation principle, namely, giving up desires, displaying compassion, and practicing meditation for seeking Buddhist wisdom. These three ways are essential ways to experience the reality of emptiness and the importance of compassion, leading to a sense of no identity. The transition from the self state to the non-self state is a deeply transformative experience of eliminating the sense of self and its psychological structures, seeing through, and overcoming the illusion of the self leading to authentic-durable happiness. Bianco et al. indicated the urgent necessity of a psychodynamic model for future development of positive psychology. Those articles provide us strong confidence that the strategy of constructing culture-inclusive theories by integrating Western and Eastern philosophies may open a new field of psychology for self-cultivation to compete with the popular positive psychology emphasizing the values of hedonism, individualism, and utilitarianism.

## Author contributions

All authors listed, have made a substantial direct and intellectual contribution to the work, and approved it for publication.

### Conflict of interest statement

The authors declare that the research was conducted in the absence of any commercial or financial relationships that could be construed as a potential conflict of interest.
